# Effect of Temperature on Chinese Rice Wine Brewing with High Concentration Presteamed Whole Sticky Rice

**DOI:** 10.1155/2014/426929

**Published:** 2014-01-06

**Authors:** Dengfeng Liu, Hong-Tao Zhang, Weili Xiong, Jianhua Hu, Baoguo Xu, Chi-Chung Lin, Ling Xu, Lihua Jiang

**Affiliations:** ^1^Key Laboratory of Industrial Advanced Process Control for Light Industry of Ministry of Education, Jiangnan University, Wuxi 214122, China; ^2^Key Laboratory of Industrial Biotechnology of Ministry of Education, Jiangnan University, Wuxi 214122, China; ^3^Shaoxing Nverhong Wine Company Limited, Shangyu, Zhejiang 312000, China

## Abstract

Production of high quality Chinese rice wine largely depends on fermentation temperature. However, there is no report on the ethanol, sugars, and acids kinetics in the fermentation mash of Chinese rice wine treated at various temperatures. The effects of fermentation temperatures on Chinese rice wine quality were investigated. The compositions and concentrations of ethanol, sugars, glycerol, and organic acids in the mash of Chinese rice wine samples were determined by HPLC method. The highest ethanol concentration and the highest glycerol concentration both were attained at the fermentation mash treated at 23°C. The highest peak value of maltose (90 g/L) was obtained at 18°C. Lactic acid and acetic acid both achieved maximum values at 33°C. The experimental results indicated that temperature contributed significantly to the ethanol production, acid flavor contents, and sugar contents in the fermentation broth of the Chinese rice wines.

## 1. Introduction

Chinese rice wine, a natural and nondistilled wine, is very popular in China and its market is speedily increasing [1]. The annual consumption is about 1.4 million tons. Hitherto, the Chinese rice wine brewing process is mainly controlled by experienced technician rather than by scientific instruments. This technician control method causes each batch of Chinese rice wine with different flavors. Currently, how to standardize all batches of Chinese rice wine with the same flavor is still an unresolved issue. Good taste becomes more important than ever for the Chinese rice wine. Young drinkers have more choices for drinks. Consequently, the wine should be with good and consistent taste to attract more customers. It is thus very important to study the effects of temperature on Chinese rice wine brewing.

Similar to sake and other rice wine varieties, the fermentation process of Chinese rice wine brewing can be divided into two stages: the main stage (also called primary fermentation) and the second stage (also called postfermentation). In the main stage, pre-steamed rice, *Saccharomyces cerevisiae* yeast culture, and wheat *qu* are mixed and fermented for 96 h [2]. During the entire process of Chinese rice wine brewing, the main stage is the core of Chinese rice wine brewing and determine the Chinese rice wine quality.

The main stage of the fermentation process is a typical simultaneous saccharification and fermentation (SSF) process as well as a semisolid state and semiliquor state fermentation (SSSLF) process. As the concentration of pre-steamed rice and wheat in mash is very high (can be as high as 45%), the SSF and SSSLF process may decrease yeast cell growth inhibition with high sugar concentration and facilitate ethanol production in Chinese rice wine brewing. The concentration of ethanol can thus be high and even more than 20% (v/v) in the final mash at the end of the main stage fermentation [3].

Temperature effects on wine fermentation have been widely investigated in beer [4], grape wine, and other ethanol fermentations [5]. Research results suggested that temperature can affect glycerol and ethanol production [6]. The effects of temperature, pH, and sugar concentration on the growth rates and cell biomass of wine yeasts were studied in grape juice wine [7]. Fermentation temperature can affect the microbial population during grape-must fermentation [8] and then affect the ethanol production of grape wine. Both yeast strain and temperature can affect the grape-wine fermentation rate and wine quality [9]. Redón et al. [10] found that temperature can affect membrane lipid composition of *Saccharomyces cerevisiae* yeast species and then affect ethanol production. In addition, appropriate pH value also is necessary for yeast growth and ethanol production [11].

The proportions of sugars, glycerol, ethanol, and organic acids are primarily responsible for the delicate taste and flavors of Chinese rice wine [12, 13]. Especially, organic acid (i.e., lactic acid) and ethanol can produce esterification in the long time storage stage and form the wine's good taste and smell. In addition, sugar contents in Chinese rice wine determine the wine types. In the National Standard of China GB 13662–2000, Chinese rice wine is divided into four types according to the concentrations of the total sugar: dry type (total sugar ≤ 15 g/L), semidry type (15 g/L < total sugar ≤ 40 g/L), semisweet type (40 g/L < total sugar ≤ 100 g/L), and sweet type (total sugar > 100 g/L).

In the past, pursuit of high ethanol concentration is the main goal for wine fermentation. At present, volatile compounds in wine have become the new important parameters to evaluate the wine quality [4, 6, 14–16]. It is clear that sugar and volatile acids can influence the taste of drink and juice [17–19]. Volatile organic acids are important to the flavor and taste characteristics of the Chinese rice wine [20]. Especially, lactic acid was the most important volatile acid [21] and constituted over 90% of the total volatile acids.

Due to the increasingly recognized importance of sugars and acids and their relationship to wine quality, it is important to investigate the effect of temperature on the yeast fermentation, organic acid, and glycerol compound during Chinese rice wine brewing. The experiment which simulated Chinese rice wine fermentation process was implemented at various temperatures (18°C, 23°C, 28°C, and 33°C) in a scale-down level. Based on previous research, 33°C is the highest temperature designed in plant fermentation process, 28°C is the desired temperature for this yeast cell growth [22], and 25°C–28°C is the desired temperature for the start of the fermentation, as 5°C was a temperature gradient and 23°C and 18°C were picked for comparison purposes [23]. As a result, these four temperatures were chosen. Natural fermentation (the surrounding temperature is 16°C and labeled as RT) was added as the control.

The results of the study contributed significantly to the understanding of the role of temperature in ethanol, organic acids, glycerol, and sugars kinetics during Chinese rice wine brewing and also provided useful information to improve the quality of Chinese rice wine.

## 2. Materials and Methods

### 2.1. Microorganisms for Fermentation

*Saccharomyces cerevisiae Su*-25 (Shaoxing Nverhong Rice Wine Plants, Zhejiang, China) was used and stored at 4°C. Chinese wheat *qu *(Shaoxing Nverhong Rice Wine Plants, Zhejiang, China) was used to hydrolyze rice starch which was stored at room temperature. Sticky rice bought from a local market (Vanguard Market, Wuxi, China) was used.

### 2.2. Small-Scale Chinese Rice Wine Brewing with Designed Experiments

#### 2.2.1. Yeast Medium and Yeast Culture

Yeast extract peptone dextrose medium (YPD) includes glucose 20 g/L, peptone 20 g/L, yeast extract 10 g/L, and agar 20 g/L.

Liquid yeast extract peptone dextrose medium (LYPD) includes glucose 20 g/L, peptone 20 g/L, and yeast extract 10 g/L.

The yeast strain was stored at 4°C on slants of yeast peptone dextrose agar medium (YPD). The yeast inoculum was transferred to a new slant of YPD and cultured for 24 h at 28°C. A sloop of yeast culture was added to 50 mL LYPD medium (250 mL flask) and cultured at 28°C for 18 h as yeast seed. The prepared yeast seed was diluted at a ratio of 1 : 10 with new LYPD (100 mL in 500 mL bottle) medium and cultured at 28°C for another 18 h.

#### 2.2.2. Batch Fermentation

Fermentation experiments were conducted in a 7 L tank fermenter (BioFlo IV, NBS Edison, NJ, USA). Sticky rice was steamed for 45 min and cooled at room temperature to 26°C. 1200 g steamed rice (dry weight), 204 g wheat *qu*, 120 mL yeast seed culture, and 2400 mL tap water were mixed in the fermenters. During the entire fermentation process, the pH was not controlled; aeration and agitation both were set at 0 value. The temperature was maintained at preset value as RT, 18°C, 23°C, 28°C, and 33°C.

The fermentation process was maintained at constant temperature as designed for 4 days. 2 mL of samples from each experiment was taken out every 2 h and centrifuged at 10000 r/min for 3 min, filtered (0.22 *μ*m, PVC membrane), and quickly analyzed with high-performance liquid chromatography (HPLC). The HPLC method was used to identify each component with the elution time and quantified by using the spiking technique.

### 2.3. Chemicals

Submicron-filtered HPLC-grade water was used. D-glucose, D-fructose, maltose, maltotriose, and sulfuric acid purchased from Sigma-Aldrich (St Louis, MO, USA) were used. HPLC-grade lactic acid, acetic acid, succinic acid, citric acid, malic acid, tartaric acid, propionic acid, ethanol, and acetonitrile purchased from Fisher (Pittsburgh, PA, USA) were used.

### 2.4. Analysis of Enological Parameters

In order to compare the effect of various fermentation conditions on the Chinese rice wine brewing process, several enological parameters were determined offline right after the samples were taken out of the fermenter.

The concentrations of sugars, glycerol, ethanol, and organic acids were determined with HPLC (Agilent 1200 series). At the predetermined time, wine samples of 1 mL were taken for analyses. Durapore (PVDF, 0.45 *μ*m pore) membrane filters (Fisher, Pittsburgh, PA, USA) were used to filter wine samples. An Aminex HPX-87H column (300 × 7.8 mm) (Bio-Rad Labs, Richmond, CA, USA) was used to determine the concentrations of the sugars, glycerol, ethanol and organic acids. A Bio-Rad HPLC column heater was used to maintain column temperature as 55°C. A Bio-Rad 125-0131 guard cartridge (Bio-Rad Labs, Richmond, CA, USA) was used to protect column. The eluted compounds (sugars, glycerol, ethanol and organic acids) were detected with an G1314B VWD detector (Agilent 1200 Series, Santa Clara, CA, USA) and an G1362A RID detector (Agilent 1200 Series, Santa Clara, CA, USA) simultaneously. The solvent delivery system was driven by a G1311A quaternary pump (Agilent 1200 Series, Santa Clara, CA, USA).

Every 1000 mL mobile phase consisted of 1270 *μ*L sulfuric acid and 60 mL acetonitrile. The samples were eluted with the mobile phase at a flow rate of 0.5 mL/min. Injection volume for each sample was 20 *μ*L per fixed loop with run time as 30 minutes.

HPLC system (Agilent 1200 Series, Santa Clara, CA, USA) was used. Separate calibration standard curves were constructed.

The statistical analysis of the final lactic acids and ethanol concentrations was performed with SAS software (version 9.3; SAS Institute, Cary, NC).

## 3. Results and Discussion

### 3.1. Effect of Temperatures on Ethanol Production in Fermentation Mash

Temperature is an important controlling parameter controlling the Chinese rice wine quality. However, it is still not clear why it affects the quality of Chinese rice wine. As ethanol is the main product of Chinese rice wine, exploring the effect of temperature on ethanol production is necessary for optimizing rice wine fermentation.

In this work, ethanol production under various temperatures (RT, 18, 23, 28, and 33°C) was investigated and presented in Table 1. It is clear that with the temperature increasing, the ethanol yield increased from 9.8% (v/v) to 12.2% (v/v) and then decreased from 12.2% (v/v) to 10.4% (v/v) and 3.6% (v/v) with the temperature increasing from 18°C to 23°C, 28°C, and 33°C, respectively. It is to be noted that the ethanol production at 11.2% (v/v) was achieved at RT condition. Ethanol production at 23°C was the highest compared to other temperatures in this work. However, Lee found that the optimal temperature for growth was 34°C, while the specific ethanol production rate was maximal at 37–43°C with *Saccharomyces uvarum *[24–26]. It is conceivable that a suitable temperature would facilitate *Saccharomyces cerevisiae Su*-25 to use sugars to produce ethanol during the Chinese rice wine brewing process. Low temperature slows down reaction rate, but excessive temperature accelerates cellular aging, and aged cells reduce ethanol production.

**Figure 1:**
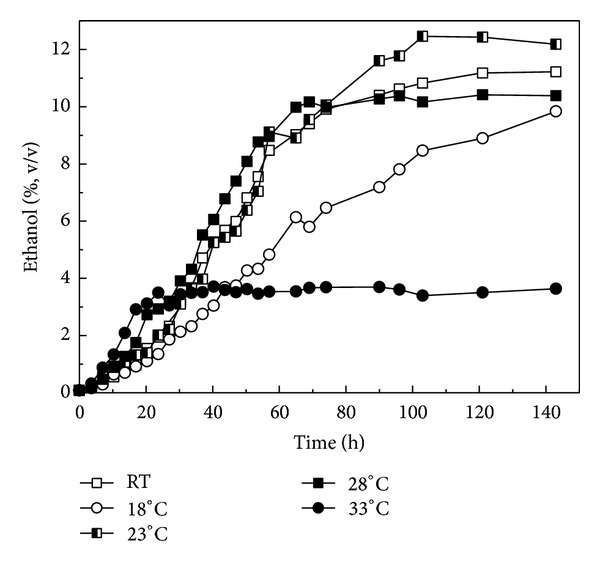
The profiles of ethanol concentration under various temperatures.

Detailed evaluation of the effect of various temperatures on ethanol production is an efficient way to analyze the kinetics of ethanol fermentation during Chinese rice wine production which are shown in Figure 1.

For the first 20 hours, the ethanol concentration increased with temperature. The highest concentration was achieved at 33°C and the lowest concentration was detected at 18°C. From 20 h to 70 h, the profiles of ethanol concentration under 23°C, 28°C, and RT were all similar. After 20 h, ethanol concentration under 33°C only has just a little fluctuation. At 18°C the ethanol concentration kept growing and was much higher than that of 33°C, but slightly lower than that at other temperatures at the 70 h. However, from 70 h to 100 h, the profile of ethanol concentration at RT and 23°C increased quickly, which only slightly increased at 28°C. From 100 h to 140 h, the ethanol concentration at 18°C increased quickly. However, under the other temperature, there were only slight variations of the ethanol concentrations. At 33°C, the ethanol concentration only slightly increased from 20 h to 140 h. All these data indicate that temperatures have different effect on ethanol production kinetics at different fermentation stages.

Ethanol production kinetics at various stages of Chinese rice wine brewing under designed temperatures was further analyzed (Table 2). At the stages of 0–47 h, highest ethanol production was achieved at 28°C. After 47 h, highest ethanol production was reached at 23°C. Consequently, a two-stage temperature controlling strategy is better for the enhancement of ethanol production in the Chinese rice wine brewing process.

### 3.2. Effect of Temperatures on Sugars and Glycerol Concentrations in Fermentation Mash during the Main Stage

Sugars in the fermentation mash of Chinese rice wine not only are important nutrient components for rice wine production but also contribute to its taste and flavor. Chinese rice wine (commonly known as Shaoxing huangjiu) is divided into four types according to its total sugar contents as shown in the Introduction. Among it, semidry rice wine is the most popular [27, 28].

**Figure 2:**
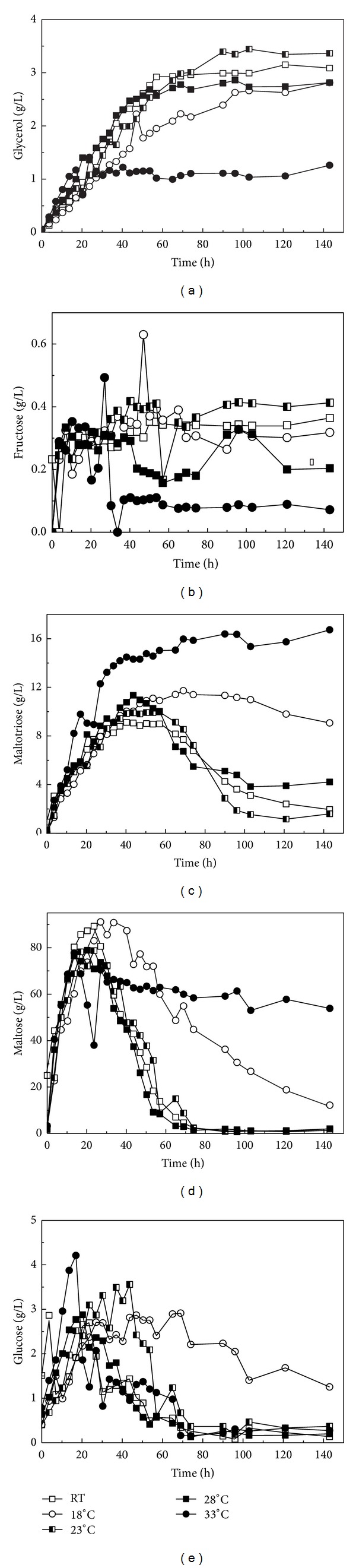
Time course of changes in productive concentration of sugars and glycerol under various temperatures: (a) glycerol, (b) fructose, (c) maltotriose, (d) maltose, and (e) glucose.

The production of sugars and glycerol in the fermentation mash of Chinese rice wine fermentation with designed experiments, including glucose, maltose, maltotriose, and glycerol, was analyzed. The fermentation profiles of sugars and glycerol are shown in Figure 2. The glycerol concentration is shown in Figure 2(a) in a similar way to the changes of ethanol level, the highest concentration of glycerol was achieved at 23°C at 3.5 g/L. For the batch tested under RT condition, the concentration of glycerol was 3.2 g/L, and for the batch at 18°C and 33°C the concentration of glycerol was 2.5 g/L and 3.5 g/L, respectively. The fermentation kinetics for fructose is shown in Figure 2(b). The highest concentration of fructose production is 0.42 g/L at 23°C followed by conditions at RT, 18°C, 28°C, and 33°C.

However, the concentrations for maltotriose at various temperatures are different from that for glycerol and fructose. The maximum concentrations of maltotriose at various temperatures were in the descending order of 33°C 18°C, 28°C, RT, and 23°C (Figure 2(c)). It is clear that all the concentrations of fructose at various temperatures have similar pattern. Fructose concentrations began to accumulate and reached maximum at 40 h, and then all the concentrations of fructose at various temperature exhibited little variations. Considering the facts that saccharification process was completed at the end of 40 h, the fructose cannot be used by *Saccharomyces cerevisiae Su*-25 and other microbial cells. The profiles of maltose concentration are shown in Figure 2(d). Similar profiles for maltose concentration to maltotriose concentration are observed under all temperature conditions tested. The highest concentration of maltose of 54.5 g/L in final fermentation mash was attained at 33°C at 140 h, and 12.4 g/L at 18°C. For other conditions, the concentrations of maltose were all low. The highest concentrations of total sugars were below 105 g/L at all temperatures during the entire process. A concentration lower than that can conceivably inhibit yeast cell growth and fermentation [29]. This experimental result agrees with previous research [2]. The glucose concentrations at different temperatures are all low and under 4 g/L during the fermentation process. The results suggest that glucose should not be the main sugar used by *Saccharomyces cerevisiae Su*-25 and other microbial cells to produce ethanol and acids during Chinese rice wine fermentation.

The fermentation kinetics of maltotriose and maltose are quite similar. This phenomenon can be explained below. Under low and high temperature, the fermentation rate is low. As maltose and maltotriose were utilized slowly by the microbial cells, both the observed residual maltose and maltotriose were higher. However, the low fermentation rate is different between low and high temperatures. The cellular metabolic activity at low temperature is generally low which conceivably caused retarded ethanol biosynthesis. On the contrary, at higher temperature, cellular aging process was accelerated which reduced ethanol formation at most of the fermentation processes. In addition, *Saccharomyces cerevisiae Su*-25 conceivably uses maltose but not glucose from which ethanol and favors were produced. This is different from that previously reported in the literature [3, 30].

### 3.3. Effect of Temperature on Organic Acid Composition in Fermentation Mash

A certain amount of acids played an important role in the flavor of rice wine and gradually converted into aromatic esters during storage. Therefore, the total acid content in the fermentation mash of Chinese rice wine is a key parameter to evaluate and control the fermentation process during industrial Chinese rice wine brewing [27]. The production of acid metabolites, including acetic acid, lactic acid, and succinic acid, was analyzed.

**Figure 3:**
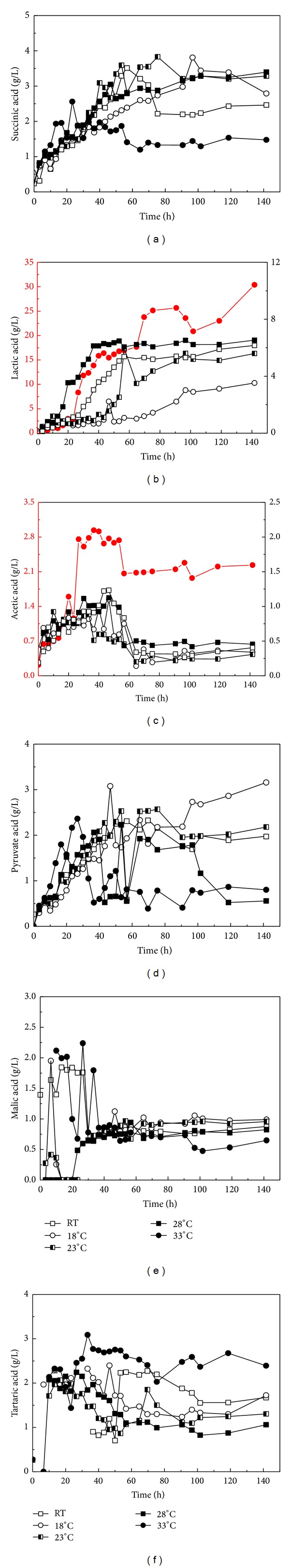
Time course of changes in the concentration of organic acids under various temperatures: (a) succinic acid, (b) lactic acid, (c) acetic acid, (d) pyruvate acid, (e) malic acid, and (f) tartaric acid.

The fermentation profiles of organic acids are shown in Figure 3. The kinetics of succinic acid fermentation at various temperatures is similar to that of ethanol production. The highest concentration of succinic acid was achieved at 23°C, and the lowest concentration was at 33°C (Figure 3(a)). The concentration of lactic acid increased quickly during the fermentation process at 33°C (Table 3). The final concentration of lactic acid is about 6 times higher compared to that under other conditions (Figure 3(b)). Similar phenomena were observed in the concentration of acetic acid, which was 2 to 5 times higher compared with the concentration of acetic acid at other temperatures (Figure 3(c)). In addition, the concentration of tartaric acid at 33°C was higher than that at other temperatures (Figure 3(f)). Propionic acid was not observed in the fermentation mash.

Only the concentrations of lactic acid and acetic acid at 33°C are statistically significantly higher than that at other temperatures which agrees with that of Mao [31]. Lactic acid was mainly produced by *Lactobacillus* during Chinese rice wine brewing [32]. At high temperature (33°C), yeast cell (*Saccharomyces cerevisiae Su*-25) growth (generally optimal at 28°C), was inhibited from producing ethanol with the substrate of glucose. Nevertheless, *Lactobacillus* can grow well to produce lactic acid from glucose at high temperature (33°C). The fermentation kinetics for succinic acid, pyruvic acid, and malic acid are similar to that of ethanol production. All the three acids are the by-products of *Saccharomyces cerevisiae Su*-25 during ethanol fermentation. This essentially agrees with previous research [23, 33].

## 4. Conclusion

Results from this study have shown that different fermentation temperatures affect the levels of sugars, glycerol concentration, and organic acid in the fermentation medium for Chinese rice wine production. The highest concentration of ethanol was achieved at 23°C. The lowest concentration of ethanol was at 33°C.

Higher temperatures can enhance organic acid production through stimulation of the growth of *Lactobacillus*. The concentrations of acetic acid, tartaric acid, and lactic acid were statistically significantly higher at 33°C than those at other temperatures (Tukey's test was used for analysis of variance to find significant differences among various treatments at *P* = 0.05 level). Lactic acid was mainly produced by *Lactobacillus*. High temperature can speed up the growth of *Lactobacillus *and production and accumulation of lactic acid.

Although it is proven that temperatures can affect the production of ethanol, glycerol, and organic acid, their optimal level in Chinese rice wine fermentation and how to accurately control their ratio through controlling temperatures are still not clear. Consequently, developing a kinetic model to describe the effect of temperatures on ethanol, glycerol, and organic acid production during Chinese rice wine fermentation is needed and is currently in progress.

## Figures and Tables

**Table 1:** Ethanol concentration (%, v/v ± SD) under various temperatures.

Temperature	RT	18°C	23°C	28°C	33°C
Ethanol (%, v/v)	11.2 ± 0.27	9.8 ± 0.12	12.2 ± 0.2	10.4 ± 0.3	3.6 ± 0.19

**Table 2:** Effect of various temperatures on the ethanol concentration (%, v/v ± SD) at different stages.

Temperature	Ethanol production (%, v/v )							
	0–14 h	14–24 h	24–37 h	37–47 h	47–57 h	57–74 h	74–96 h	96–140 h
RT	0.83 ± 0.04	1.02 ± 0.02	2.77 ± 0.21	1.28 ± 0.05	2.49 ± 0.08	1.44 ± 0.11	0.7 ± 0.08	0.6 ± 0.09
18°C	0.61 ± 0.00	0.65 ± 0	1.4 ± 0.22	0.99 ± 0.07	1.08 ± 0.05	1.64 ± 0	1.34 ± 0.11	2.04 ± 0.21
23°C	0.98 ± 0.03	0.96 ± 0.01	1.95 ± 0.01	1.68 ± 0.04	3.47 ± 0.33	0.94 ± 0.04	1.72 ± 0.08	0.41 ± 0.1
28°C	1.18 ± 0.00	1.66 ± 0.03	2.58 ± 0.16	1.88 ± 0.13	1.57 ± 0.09	1.01 ± 0.06	0.41 ± 0.02	0.01 ± 0.03
33°C	2 ± 0.06	1.41 ± 0.03	0.02 ± 0.16	0 ± 0.03	0.02 ± 0.03	0.15 ± 0.04	−0.07 ± 0.04	0.02 ± 0.03

**Table 3:** Effect of changing temperature on lactic acid concentration (g/L ± SD) at different stages.

Temperature	Lactic acid concentration (g/L)							
	0–14 h	14–24 h	24–37 h	37–47 h	47–57 h	57–74 h	74–96 h	96–140 h
RT	0.5 ± 0.2	0.25 ± 0.03	1.92 ± 0.01	1.13 ± 0.08	1.2 ± 0.07	−0.18 ± 0.09	0.12 ± 0.04	0.85 ± 0.02
18°C	0.57 ± 0.31	−0.03 ± 0.01	0.12 ± 0.03	1.57 ± 0.03	−1.15 ± 0.01	0.36 ± 0.01	1.58 ± 0.03	0.5 ± 0.06
23°C	0.61 ± 0.11	−0.06 ± 0.01	0.22 ± 0.03	0.67 ± 0.03	4.41 ± 0.01	−1.6 ± 0.01	1.25 ± 0.03	−0.01 ± 0.06
28°C	1.08 ± 0.28	2.38 ± 0.04	2.56 ± 0.00	0.09 ± 0.00	−0.17 ± 0.02	0 ± 0.01	0.46 ± 0.03	0.01 ± 0.01
33°C	0.86 ± 0.01	1.81 ± 0.07	11.05 ± 0.79	1.58 ± 0.15	1.47 ± 0.26	8.21 ± 0.22	−1.55 ± 0.59	6.79 ± 0.06
